# Immunotherapy Based on Dendritic Cell-Targeted/-Derived Extracellular Vesicles—A Novel Strategy for Enhancement of the Anti-tumor Immune Response

**DOI:** 10.3389/fphar.2019.01152

**Published:** 2019-10-11

**Authors:** Oleg Markov, Anastasiya Oshchepkova, Nadezhda Mironova

**Affiliations:** Laboratory of Nucleic Acids Biochemistry, Institute of Chemical Biology and Fundamental Medicine SB RAS, Novosibirsk, Russia

**Keywords:** dendritic cells, extracellular vesicles, exosomes, tumor, anti-tumor vaccines

## Abstract

Dendritic cell (DC)-based anti-tumor vaccines have great potential for the treatment of cancer. To date, a large number of clinical trials involving DC-based vaccines have been conducted with a view to treating tumors of different histological origins. However, DC-based vaccines had several drawbacks, including problems with targeted delivery of tumor antigens to DCs and prolong storage of cellular vaccines. Therefore, the development of other immunotherapeutic approaches capable of enhancing the immunogenicity of existing DC-based vaccines or directly triggering anti-tumor immune responses is of great interest. Extracellular vesicles (EVs) are released by almost all types of eukaryotic cells for paracrine signaling. EVs can interact with target cells and change their functional activity by delivering different signaling molecules including mRNA, non-coding RNA, proteins, and lipids. EVs have potential benefits as natural vectors for the delivery of RNA and other therapeutic molecules targeted to DCs, T-lymphocytes, and tumor cells; therefore, EVs are a promising entity for the development of novel cell-free anti-tumor vaccines that may be a favourable alternative to DC-based vaccines. In the present review, we discuss the anti-tumor potential of EVs derived from DCs, tumors, and other cells. Methods of EV isolation are systematized, and key molecules carried by EVs that are necessary for the activation of a DC-mediated anti-tumor immune response are analyzed with a focus on the RNA component of EVs. Characteristics of anti-tumor immune responses induced by EVs *in vitro* and *in vivo* are reviewed. Finally, perspectives and challenges with the use of EVs for the development of anti-tumor cell-free vaccines are considered.

## Dendritic Cells: A Brief History of Anti-Tumor DC-Based Vaccine Development

The history of dendritic cells (DCs) began during the second half of the nineteenth century, when Paul Langerhans described for the first time star-shaped cells localized in the skin and mistakenly assumed a neuronal origin (Langerhans, 1868). Eventually, these cells were named Langerhans cells and were found to be a special skin resident subpopulation of DCs (Merad et al., 2008). DCs were rediscovered by Ralph M. Steinman and Zanvil A. Cohn a century later in 1973 (Steinman and Cohn, 1973), and this discovery eventually launched a new era in immunology. It was found that DCs are professional antigen-presenting cells, the main function of which is to capture, process, and present antigen material to T lymphocytes in complex with MHC I and II molecules, activating an antigen-specific T-lymphocyte immune response (Nussenzweig et al., 1980). It was understood how the antigen-specific T-cell immune response is triggered, and DCs were shown to play a principal role in this process (Dhodapkar et al., 1999).

Later, the method of generating a large number of DCs was developed on the basis of the incubation of monocyte and bone marrow DC progenitors in the presence of IL-4 and GM-CSF (Romani et al., 1994; Sallusto et al., 1995), which significantly facilitated investigations into the biology and immunotherapeutic potential of DCs. As a result of these studies, a novel type of immunotherapeutic anti-tumor vaccine was developed, namely, the DC-based vaccine (Constantino et al., 2016). Classically, this vaccine is based on DCs loaded with tumor antigens using different approaches ranging from passive loading of DCs with tumor proteins or peptides to transfection/transduction of DCs with nucleic acids (NAs)/viral vectors encoding tumor antigens (Palucka and Banchereau, 2013).

Several decades of intensive investigations of anti-tumor DC-based vaccines have revealed their high efficiency in various murine tumor models (Lin et al., 2015; Markov et al., 2017) and human xenografts in immunodeficient mice (Liu et al., 2019).

Now in clinical trials a great number of DC-based vaccines are explored (see revs. Van Willigen et al., 2018; Mastelic-Gavillet et al., 2019). The first therapeutic anti-tumor DC-based vaccine, Sipuleucel-T, was approved by the U.S. Food and Drug Administration in 2010 for use in castration-resistant prostate tumors. Sipuleucel-T activates the immune response against the antigen, PAP/PA2024, and increases overall survival in patients (Kantoff et al., 2010); however, Sipuleucel-T is highly priced cellular product with technology challenging preparation process which required highly qualified personnel (Van Willigen et al., 2018).

The Indian government agency (CDSCO-Central Drugs Standard Control Organization) approved in 2017 vaccine based on an autologous monocyte-derived DC loaded with tumor lysate (APCEDEN®) for treatment of prostate, ovarian and colorectal cancers, and non–small cell lung carcinoma (Kumar et al., 2017). In phase II clinical trial this vaccine demonstrated well tolerance by patients with refractory solid malignancies (Bapsy et al., 2014) and a survival benefit over 100 days (Kumar et al., 2017).

Current ongoing clinical trials using personalized DC-based vaccines are conducted for treatment of ovarian cancer; brain tumors; advanced melanoma, colorectal cancer, lung cancer, and so on (**Table 1**). Types of vaccines studied in clinical trials are varied by the composition of DC-pulsed antigen that can be in the form of tumor lysate, tumor-derived peptides, and mRNA-encoding TAAs. Some types of DC-vaccines are prepared by fusion of DC with tumor cells. Introduction in the treatment regimen of additional components including chemotherapeutics (NCT01957956, 2013; NCT01946373, 2013; NCT02503150, 2015; **Table 1**), immune response modifiers (NCT01808820, 2013; NCT01204684, 2010; NCT00799110, 2008; **Table 1**), and immune checkpoint inhibitors (NCT03014804, 2017; NCT02529072, 2015; NCT02678741, 2016; NCT03092453, 2017; NCT03152565, 2017; NCT03406715, 2018; **Table 1**), as well as cytokines providing the maturation of DC lead to the novel treatment schemes with enhanced efficacy. DC-based vaccines demonstrate their high potential, and some of them have already reached phase 3 of clinical trials: DC-vaccine loaded with tumor-derived RNA for treatment of uveal melanoma (NCT01983748, 2013, **Table 1**) and tumor-lysate pulsed DC-vaccine in combination with FOLFOX6 regiment for treatment of metastatic colorectal cancer (NCT02503150, 2015; **Table 1**).

**Table 1:** Ongoing clinical trials of dendritic cell-based vaccines for treatment of various types of tumors.

Type of tumor	Type of TAA pulsed to DC	Vaccine composition	Primary / secondary outcomes	Sponsor	Phase	NCT identifier
*Brain tumors*						
newly diagnosed glioblastoma	tumor lysate	DC-vaccine/ temozolomide*	Toxicity/ Clinical benefit rate, duration of response, overall response rate	Mayo Clinic, National Cancer Institute (NCI), USA	early phase 1	NCT01957956, 2013
recurrent glioblastoma	tumor lysate	DC-vaccine	Toxicity/ Clinical benefit rate, duration of response, overall response rate, OS, PFS, time to response		early phase 1	NCT03360708, 2017
malignant glioma/glioblastoma,	tumor lysate	DC-vaccine/ Imiquimod**	Adverse events/ OS, PFS, IR	Macarena De La Fuente, University of Miami, USA	Phase 1	NCT01808820, 2013
glioma/ astrocytoma/ astrodendroglioma/ glioblastoma	tumor lysate	DC-vaccine + 0.2% resiquimod**/ autologous DC-vaccine + poly ICLC^#^	Most effective combination of DC vaccine components/ Time to tumor progression, OS	Jonsson Comprehensive Cancer Center, USA	Phase 2	NCT01204684, 2010
recurrent glioblastoma	tumor lysate	DC-vaccine/Nivolumab^$^	Adverse events, OS/ PFS, QoL, CR, PR, SD, PD, RSDR		Phase 2	NCT03014804, 2017
recurrent malignant glioma/ astrocytoma/ glioblastoma	CMV pp65-LAMP mRNA	DC-vaccine/Nivolumab^$^	Safety/ OS, PFS	Gary Archer Ph.D., Duke University, USA	Phase 1	NCT02529072, 2015
glioblastoma		DCs/ autologous lymphocytes/ tetanus toxoid	Feasibility, safety/ humoral and cellular IR, time to progression		Phase 1	NCT00639639, 2008
*Melanoma*						
malignant melanoma	tumor lysate	DC-vaccine/ T-cells/ cyclophosphamide*/ fludorabine*	Safety/ Time to disease progression	Karolinska University Hospital, Sweden	Phase 1	NCT01946373, 2013
metastatic melanoma	tumor lysate	TLPLDC-vaccine^@^/ Checkpoint inhibitors	Safety/ Tumor response to treatment	Cancer Insight, LLC, USA	Phase 1/ Phase 2	NCT02678741, 2016
melanoma		TLPLDC-vaccine^@^	Disease free survival		Phase 2	NCT02301611, 2014
malignant melanoma Stage III/ Stage IV	tumor lysate	DC-vaccine/RT/IFN-α	Safety, tolerability, feasibility, irDCR, IR / OS, irTTP, irORR, irDOR, irTTR, irPFS	Istituto Scientifico Romagnolo per lo Studio e la cura dei Tumouri, Italy	Phase 2	NCT01973322, 2013
		DC-vaccine	RFS/ OS, IR		Phase 2	NCT02718391, 2016
advanced melanoma	tumor-derived peptide	DC-vaccine/ Pembrolizumab^$^/ cyclophosphamide*	IR/ Clinical response, time to progression, safety, adverse events	University of Pennsylvania, USA	Phase 1	NCT03092453, 2017
uveal melanoma	tumor-derived RNA	DC-vaccine	Prolongation of disease free survival/ OS, IR	University Hospital Erlangen, Germany	Phase 3	NCT01983748, 2013
Colorectal cancer						
colorectal cancer	tumor-associated antigen CEA	DC vaccine	Safety, feasibility/ Antigen-specific IR, pathological responses, disease-free survival	Radboud University, Netherlands	Phase 1/ Phase 2	NCT01885702, 2013
metastatic colorectal cancer	tumor lysate	DC-vaccine/ FOLFOX6^§^	PFS/ Objective response, OS, QoL, adverse events	Second Military Medical University, China	Phase 3	NCT02503150, 2015
colorectal cancer	tumor lysate	DC-vaccine/ Avelumab^$^	Dosed of Avelumab and DCs, PFS/ Adverse events, immunophenotype of tumors, MSS, RAS and BRAF mutation status	Grupo Espanol Multidisciplinario del Cancer Digestivo, Spain	Phase 1/ Phase 2	NCT03152565, 2017
*Ovarian cancer*						
ovarian cancer	tumor lysate	DC vaccine /ontak^&^	IR/ Toxicity	Loyola University, USA	Phase 2	NCT00703105, 2008
ovarian cancer primary peritoneal cancer fallopian tube cancer	–	DC-tumor fusion vaccine/ GM-CSF, imiquimod**	IR/ Toxicity, clinical response	Beth Israel Deaconess Medical Center, Israel	Phase 2	NCT00799110, 2008
*Lung cancer*						
Small-cell lung cancer	–	DC endogenously expressed p53 gene/ Ipilimumab, Nivolumab^$^	DCR/ PFS, OS, ORR, IR	H. Lee Moffitt Cancer Center and Research Institute	Phase 2	NCT03406715, 2018
Non-small cell lung cancer^©^	peptides PRS pan-DR, MAGE-3 DP04, MAGE-1 A2, MAGE-3 A2, NY-ESO-1 A2 et MART-1 A2	DC-derived exosomes	PFS	Gustave Roussy, Cancer Campus, Grand Paris, France	Phase 2	NCT01159288, 2010

*chemotherapeutic; **immune response modifier; ^#^poly ICLC, interstitial Cajal-like cells, TLR3 agonist; ^$^monoclonal antibodies, immune checkpoint inhibitor; ^&^anti CD25 denileukin diftitox; ^§^FOLFOX6 - a specific chemotherapy regimen of Oxaliplatin, 5-Fluorouracil and Leucovorin; ^©^the study has been completed; ^@^TLPLDC-vaccine - autologous tumor lysate, particle-loaded, dendritic cell vaccine;

OS, overall survival; PFS, progression free survival; QoL, quality of life; CR, number of participants with complete response; PR, number of participants with partial response; SD, number of participants with stable disease; PD, number of participants with progressive disease; RSDR, response/stable disease rate; RFS, relapse-free survival; irDCR, immune related disease control rate; irTTP, immuno-related time to progression; irORR, immuno-related overall response rate; irDOR, immuno-related duration of response; irTTR, immuno-related time to response; irPFS, immuno-related progression free survival; DCR, disease control rate; ORR, overall response rate; IR, immune response; RT, radiation treatment.

The era of investigation of DC-derived membrane vesicles for immunotherapy of cancer is just beginning, and ongoing clinical trials are extremely scarce. For instance, a clinical trial of DC-derived extracellular vesicles (EVs) for treatment of patients with non-small cell lung cancer (NSCLC) is now completed (NCT01159288, 2010, **Table 1**). It was shown that DC-derived EVs exerted natural killer (NK) cell effector functions in patients with NSCLC, thus boosting the NK cell arm of antitumor immunity (Besse et al., 2016). These findings indicate the efficiency of novel immunotherapeutic anti-tumor approaches based on membrane vesicles and their great therapeutic prospects.

## EVs as Alternative Cell-Free Anti-Tumor Vaccines

One of anti-tumor immunotherapeutic approaches is the application of EVs of DCs and tumor cells. All types of eukaryotic cells produce nano-sized vesicles with the capacity to shuttle NAs, proteins, and lipids to other cells and participate in cell-to-cell communication, thus realizing paracrine regulation. EVs are a heterogeneous population of membrane vesicles that are classified into three major groups according to their subcellular origin and size: apoptotic bodies, microvesicles (MVs), and exosomes (Gurunathan et al., 2019), the latter of which are the most studied. Exosomes are nano-sized vesicles originating from multivesicular bodies (MVBs) in the endosomal pathway, with sizes ranging from 50 to 150 nm in diameter and membranes characterized by a high content of cholesterol and glycosphingolipids (Colombo et al., 2014). MVs are described as 100- to 1000-nm vesicles enveloped from the cell surface membrane by direct budding (Morel et al., 2011). Since there exists no perfect method to isolate only exosomes (Chulpanova et al., 2018), studies on the functional activity of EVs have been performed on exosome- or MV-enriched populations or a mixture of both types of EVs. Therefore, in the present review, the common term EVs will be used to describe primarily exosome-enriched vesicles.

It has been demonstrated that EVs can participate in immune regulation, matrix remodeling, signaling pathways, intercellular exchange with oncoproteins and oncogenes, induction of angiogenesis, and preparation of a pre-metastatic niche (Lee et al., 2011). It is known that DC-derived EVs carry functionally active molecules on their surfaces that take part in immunological synapses—complexes of MHC class I and II with tumor antigens, as well as co-stimulatory and adhesion molecules (such as CD80, CD86, and CD40)—needed for the induction of anti-tumor T-cell immune responses (Munich et al., 2012). In addition, tumor cell-derived EVs have been shown to have an immunostimulatory effect on anti-tumor DCs (André et al., 2002; Liu et al., 2018); hence, the application of both DC- and tumor cell-derived EVs as novel immunotherapeutic cell-free anti-tumor vaccines has great potential. Together with their highly therapeutic anti-tumor potential, cell-free vaccines based on EVs have advantages over classical DCs involved: (1) EV-based vaccines can be stored for a prolonged time without loss of immunotherapeutic activity (Jeyaram and Jay, 2018); (2) the more efficient capture of EVs rather than the soluble molecules of antigen-presenting cells (Zeelenberg et al., 2008). Undoubtedly, the use of EVs as anti-tumor vaccines possesses great potential and relevance (Viaud et al., 2010; Pitt et al., 2016).

## Methods of EV Isolation

The most commonly described method in the literature for the isolation of EVs is sequential centrifugation of conditioned medium samples or biological fluids (blood serum, urine, milk, etc.) (Petersen et al., 2014). Typically, low speeds with increasing centrifugal force are used to remove cells, cell debris, and large particles, followed by ultracentrifugation at 100,000*g* to 120,000*g* for at least 60 to 120 min to precipitate EVs (see studies in **Table 2**). This method is relatively laborious, time-consuming, and requires special expensive equipment (Soung et al., 2017). The high heterogeneity of EVs and overlapping size with protein aggregates, as well as the need for several rounds of ultracentrifugation during the wash steps of EVs, inevitably results in EV loss, contamination, and low yields (Li et al., 2017). The widely used ultracentrifugation method for EV isolation results in the lowest recovery of particles; nevertheless, it is the most popular approach to date (Tang et al., 2017).

**Table 2:** Efficiency of antitumour vaccines on the base of tumour cell-/DC-derived EVs in animal tumour models *in vivo*, human cells *ex vivo* and in clinical trials.

A. Vaccines on the base of tumour cell-derived DC-targeted EVs.						
EV origin	Isolation method		Carried/loaded molecules	Strategy of loading	Biological outcome	Reference
Murine H22 hepatocarcinoma B16 melanoma CT26 colon carcinoma	Sequential centrifugation		DNA fragments		***Ex vivo*** Tumour-derived MVs were efficiently captured by DCs, stimulated DCs via cGAS/STING signaling → activation of type I IFN production by DCs → DC maturation and presentation of tumour antigens to T cells. ***In vivo*** **H22 hepatocarcinoma, B16 melanoma, CT26 colon carcinoma** Vaccination (SC) of mice with microvesicles (MVs) activated protective immune response against tumours. Vaccination (SC) of mice with DCs loaded with MVs activated efficient antitumour therapeutic response. MVs were more immunogenic in comparison with exosomes	Zhang et al., 2014a
Murine leukemia L1210 Human leukemia K562	Sequential centrifugation				***Ex vivo*** DCs loaded with tumour-derived EVs (DC/EVs) induced antileukemic CTLs more efficiently than EVs alone. ***In vivo*** **L1210 leukemia model** Prophylactic vaccination (SC) of mice with DC/EVs provided protection of 70-100% of mice against tumour development. Therapeutic vaccination (SC) of tumour-bearing mice with DC/EVs resulted in complete regression of tumours.	Yao et al., 2014
Human glioma	Sucrose centrifugation				***Ex vivo*** DCs/EVs activated glioma-specific CTLs *ex vivo* that lysed glioma cells 2-fold more efficiently as compared to tumour lysate-pulsed DCs.	Bu et al., 2011
Murine malignant mesothelioma AB1	Sequential centrifugation				***In vivo*** **AB1 mesothelioma model** Vaccination of tumour-bearing mice with DC/EVs increased median and overall survival as compared to vaccination with tumour lysate-loaded DCs (median survival 29.5 or 18.5 days, respectively; overall survival 33.3% or 16.7%, respectively).	Mahaweni et al., 2013
Murine myeloid leukemia WEHI3B	Sequential centrifugation				***In vivo*** **WEHI3B myeloid leukemia model** DC/EVs vaccination of tumour-bearing mice resulted in more significant retardation of tumour growth and survival in animals as compared with tumour lysate-loaded DCs. DC/EVs stimulated trogocytosis and proliferation of CD4^+^ T cells.	Gu et al., 2015
Murine EG7-OVA lymphoma	Sequential centrifugation				***In vivo*** **BL6-10-OVA melanoma model** Prophylactic vaccination (IV) of mice: EVs – 4/8 mice with tumours, 29 metastases DC/EVs – 1/8 mice with tumours, 5 metastases DC/OVA – 0/8 mice with tumours, 0 metastases. Immune stimulatory effects of EVs were depended on host DCs whereas DC/EVs were not.	Yao et al., 2013
Murine 3LL Lewis lung carcinoma	Sequential centrifugation with sucrose gradient centrifugation		CD40L	Indirect	***Ex vivo*** CD40L-EVs were more immunogenic as compared with unmodified EVs: CD40L-EVs induced ↑ mature DC phenotype and ↑ levels of IL-12 and TNF-α synthesis by DCs. DC/CD40L-EVs induced ↑ proliferation of allogeneic T cells. CD40L-EVs induced ↑ proliferation of tumour antigen-specific CD4^+^ T cells. ***In vivo*** **3LL Lewis lung carcinoma model** Retardation of tumour growth; overall survival: A. Prophylactic vaccination (SC). CD40L-EVs – in 16 times; 80%. EVs – in 3 times; 50%. B. Therapeutic vaccination (SC). CD40L-EVs – in 10 times; 50%. EVs – in 1.7 times; 0%.	Wang et al., 2014
Human A549 non-small cell lung cancer	Sequential centrifugation		Rab27	Indirect	***Ex vivo*** Rab27-EVs upregulated MHC II, CD80, CD86 on DCs and stimulated synthesis of IL-1β, TNF-α and RANTES by DCs. DC/Rab27-EVs significantly ↑ CD4^+^T cell proliferation. ***In vivo*** **A549 xenograft model** Rab27-EVs significantly ↓ tumour growth in both prophylactic and therapeutic settings in comparison with EVs (SC vaccination). Rab27-EVs activated Th1 immune response – splenocytes of mice vaccinated with Rab27-EVs expressed ↑ levels of IL-2 and IFN-γ.	Li et al., 2013
Murine L1210 leukemia cells	Sequential centrifugation		shRNA-TGF-β1	Indirect	***Ex vivo*** shRNA-TGF-β1-EVs promote maturation of DCs via ↓ TGF-β1 expression. DC/shRNA-TGF-β1-EVs more efficiently promoted: 1) CD4^+^ T cell proliferation; 2) Th1 cytokine secretion; 3) Efficient antileukemia CTL response; as compared with DC/EVs. ***In vivo*** **L1210 leukemia model** Vaccination (SC) of mice with DC/shRNA-TGF-β1-EVs in both prophylactic and therapeutic settings resulted in more significant inhibition of tumour growth in comparison with DC/EVs.	Huang et al., 2017
Murine TSA breast carcinoma cells (irradiated)	Sequential centrifugation with sucrose gradient centrifugation		Degrading cytosoli dsDNA	Indirect	***Ex vivo*** dsDNA-EVs stimulated maturation of DCs – ↑ CD40, CD80, CD86 expression and STING-dependent activation of IFN-β synthesis. ***In vivo*** **TSA breast carcinoma model** Prophylactic vaccination (SC) dsDNA-EVs elicited tumour-specific CD8^+^ T cell response that more significantly protected mice from tumour development (2/6 mice without tumours, tumours infiltrated with tumour antigen-specific CD8^+^ T cells) in comparison with EVs (0/6 mice without tumours).	Diamond et al., 2018
Murine B16BL6 melanoma	Sequential centrifugation		pH-sensitive fusogenic GALA peptide	Indirect	***Ex vivo*** GALA-EVs efficiently delivered EV cargo to the cytosol of DCs. DC/GALA-EVs showed ↑ tumour antigen presentation by MHC class I molecules.	Morishita et al., 2017
Murine B16BL6 melanoma	Sequential centrifugation		SAV-LA	Indirect	***Ex vivo*** SAV-LA-EVs + biotinylated CpG-DNA → CpG-EVs DC2.4/CpG-EVs – ↑ synthesis of TNF-α, IL-6, IL-12p40; ↑ tumour antigen presentation. ***In vivo*** **B16BL6 melanoma model** CpG-EVs vaccination (ID or IT) resulted in ↑ antitumour immune response as compared with EVs+CpG-DNA: Prophylactic settings – 3.7 times ↑ retardation of tumour growth. Therapeutic settings – 3.3 times ↑ retardation of tumour growth. Survival – 10 days ↑. Number of lung metastases – 6 times ↓.	Morishita et al., 2016
Murine 3LL Lewis lung carcinoma	Sequential centrifugation		CCL2, CCL3, CCL4, CCL5, CCL20		Heat stressed EVs (HS-EVs). HS-EVs chemoattracted CD11^+^ DCs and CD4^+^/CD8^+^ T cells *in vitro* and *in vivo.* ***In vivo*** **3LL Lewis lung carcinoma model** IT injection of HS-EVs induced more efficient specific antitumour response in comparison with EVs - ↓ tumour growth, ↑ survival.	Chen et al., 2011
Human gastric adenocarcinoma (from ascites)	Sequential centrifugation Sucrose centrifugation		HSP60, HSP70		**Heat stress improved the immunogenicity of EVs**. HS-EVs promote DC maturation, ↑ IL-12p70 and TNF-α by DCs in comparison with EVs. HS-EVs activated proliferation of T cells. HS-EVs induced tumour-specific CTL response *in vitro*	Zhong et al., 2011
Mouse MC38 colon cancer	Sequential centrifugation Sucrose centrifugation		HSP70		DCs treated with HS-EVs stimulated conversation of Treg to Th17 cells. ***In vivo*** **MC38 colon cancer model** Therapeutic settings IT vaccination of mice with HS-EVs →HSP70 on HS-EVs stimulated secretion of IL-6 by DCs → IL-6 blocked differentiation of TGF-β-induced Treg cells and promotes Th17 cell differentiation (↑IL-17 expression) *in vitro* and *in vivo* → IL-17 causes rejection of established prostate tumours in mice. Inhibition of tumour growth by HS-EVs is completely dependent upon IL-6 and partially upon IL-17. **Colorectal cancer patients** In colorectal cancer patients treated with hyperthermia Tregs conversed to Th17 (in blood serum higher serum levels of IL-6 and IL-17; ↑Th17 cells and ↓Tregs in PBMC after hyperthermia).	Guo et al., 2018
B. Vaccines on the base of DC-derived EVs.						
EV origin	Isolation method		Carried/loaded molecules	Strategy of loading	Biological effects	Reference
Murine DC2.4 Bone marrow-derived DCs (BM-DCs)	Sequential centrifugation		α-fetoprotein (AFP)	Indirect	***In vivo*** **Hepa1-6 and autochtonous hepatocellular carcinoma (ectopic, orthotopic, carcinogen-induced) models** AFP-EVs activated efficient tumour antigen-specific immune response that causes: ↑ retardation of tumour growth; ↑ overall survival. AFP-EVs reshaped tumour immune microenvironment: ↑ IFN-γ-expressing CD8^+^ T cells; ↑ levels of IFN-γ and IL-2; ↓ CD25^+^Foxp3^+^ Treg cells; ↓ levels of IL-10 and TGF-β.	Lu et al., 2017
Murine BM-DCs	Sequential centrifugation		E7_49-57_ peptide (HPV early antigen 7)	Indirect	Poly(I:C) dramatically increased the potent antitumour immunity induced by E7_49-57_-EVs. ***Ex vivo*** E7_49-57_-EVs efficiently induced anti-TC1 cervical cancer CTLs, activated proliferation of CD8^+^ T cells and IFN-γ synthesis. ***In vivo*** **TC-1 cervical cancer model** Prophylactic and therapeutic settings E7_49-57_-EVs vaccination (IV) resulted in activation of antitumour immune response in both treatment regimens. poly(I:C)-E7_49-57_-EVs vaccination markedly inhibited tumour growth and improved the overall survival rate (60% of mice).	Chen et al., 2018
Murine BM-DCs	Sequential centrifugation		Chaperone-rich lysate of GL261 glioma cells (CRCL-GL261)	Indirect	***Ex vivo*** DC/CRCL-GL261-EVs promote proliferation of CD4^+^ and CD8^+^ T cells and anti-GL261 glioma CTL activity as compared with DC/GL261-EVs. ***In vivo*** **GL261 glioma model** Therapeutic settings Vaccination of tumour-bearing mice with DC/CRCL-GL261-EVs: ↑ survival of tumour-bearing mice and ↓ tumour growth; ↑ infiltration of CD4^+^ and CD8^+^ T cells into intracranial glioma tissues; ↑ production of IL-2 and IFN-γ. Depletion of CD4^+^ and CD8^+^ T cells significantly ↓ antitumour effect of DC/CRCL-GL261-EVs. DC/CRCL-GL261-EVs downregulated Cbl-b and c-Cbl signaling in T cells → activation of PI3K/Akt and ERK signaling in T cells.	Bu et al., 2015
Human umbilical cord blood-derived DCs	Sequential centrifugation		Total tumour RNA and tumour lysate (human gastric adenocarcinoma BGC823)	Indirect	***Ex vivo*** DCs transfected with total RNA isolated from BGC823 human gastric adenocarcinoma cells and EVs from these DCs stimulated more potent proliferation of T cells and antitumour CTLs in comparison with tumour lysate-loaded DCs and EVs. ***In vivo*** **BGC823 human gastric adenocarcinoma xenograft model** Therapeutic settings (PT vaccination) DC/tumour RNA, DC/tumour lysate, tumour RNA-EVs and tumour lysate-EVs injected together with T cells significantly ↓ tumour growth in 6.7 times in comparison with unvaccinated group of mice.	Guan et al., 2014
Murine BM-DCs	Sequential centrifugation		HER2/neu-MHC I HER2-MHC I	Indirect	***In vivo*** **Tg1-1 breast cancer, HER2** **^+^** **B16 melanoma and BT474** **_A2_** **breast cancer models** Immunization (IV) of mice with CD4^+^ T cells/neu-EVs stimulated efficient neu-specific CTL response resulted in protective immunity against neu-expressing Tg1-1 breast cancer (6/6 transgenic FVBneuN mice). Immunization (IV) of mice with CD4^+^ T cells/HER2-EVs induced HER2-specific CTL response (6/6 mice) and protective immunity against HLA-A2^+^HER2^+^ BL6-10_A2/HER2_ B16 melanoma (2/8 transgenic mice – full protection, 6/8 mice – significantly prolonged survival). CD4^+^ T cells/HER2-EVs vaccine stimulated CTLs killing trastuzumab-resistant BT474_A2_ breast cancer *in vitro* and eradicating 6-day palpable HER2^+^ BT474_A2_ *in vivo* (in athymic nude mice, IV immunization).	Wang et al., 2013
Murine BM-DCs	Sequential centrifugation		OVA SIINFEKL	Indirect	***In vivo*** **B16-OVA melanoma model** Prophylactic settings CD8^+^ T cell response was induced in mice immunized (IV) with OVA-EVs (but not with SIINFEKL-EVs). CTL response activated with OVA-EVs was totally dependent on CD4^+^ T cells and additionally on B cells (full OVA protein contains both Th and B cell epitopes). OVA-EVs were superior in protecting mice against B16-OVA melanoma growth in comparison with SIINFEKL-EVs.	Naslund et al., 2013
Murine BM-DCs	Sequential centrifugation		OVA	Indirect	***Ex vivo*** OVA-Exosomes and OVA-MVs were equally enriched in immunostimulatory molecules (MHC I, MHC II, CD40, CD80, CD86, CD54). OVA-Exosomes were more potent to induce OVA-specific immune response. OVA-Exosomes carried high levels of OVA, MVs contained barely detectable OVA. OVA-Exosomes or OVA-Exosomes+OVA-MVs induced OVA-specific CD8^+^ T cells whereas OVA-MVs did not activated OVA-specific CTLs. OVA-Exosomes were more efficient in eliciting OVA-specific IgG production.	Wahlund et al., 2017
Human monocyte-derived DCs	Sequential centrifugation				***Ex vivo*** Large EVs (lEVs) and small EVs (sEVs) induced CD4^+^ T cell activation with equal efficiency. lEVs and sEVs isolated from immature DCs were different in their capacity to orient Th response: lEVs induced secretion of Th2 cytokines (IL-4, IL-5, IL-13), sEVs – Th1 cytokines (IFN-γ). EVs transmembrane receptors involved in T cell regulation – lEVs – CD80, sEVs – CD40 and DC-SIGN. lEVs and sEVs isolated from mature DCs – functional differences were abolished, both types of EVs induced IFN-γ synthesis.	Tkach et al., 2017
Murine BM-DCs	Sequential centrifugation		α-galactosylceramide (αGC), OVA	Indirect	(αGC+OVA)-EVs induced strong innate and OVA-specific adaptive immune response. (αGC+OVA)-EVs were induced γδ T cell-dependent, iNKT cell-mediated and OVA-specific T and B cell mediated immunity. ***In vivo*** **B16-OVA melanoma model** Therapeutic vaccination (IV) of mice with (αGC+OVA)-EVs resulted in: ↓ tumour growth ↑ tumour infiltration with antigen-specific CD8^+^ T cells, ↓ median survival In comparison with melanoma-bearing mice immunized with soluble αGC and OVA. Boosting vaccination with (αGC+OVA)-EVs further ↑ effects of treatment.	Gehrmann et al., 2013
Human monocyte-derived DCs Murine BM-DC	Ultrafiltration/ diafiltration		MAGE.A1 and MAGE3.DP04 (human EVs)	Indirect Direct	Mouse EVs promoted IL15Rα-dependent proliferation and NKG2D-dependent activation of NK cells *in vivo.* Human EVs carried functional IL-15Rα and transpresented IL-15 to NK with activating proliferation of NK and production of IFN-γ *in vitro.* Human EVs carried NKG2D ligands (ULBP-1, MICA/B) that promoted activation of NK cells.	Viaud et al., 2009
Murine BM-DCs	Sequential centrifugation		TNF, FasL, TRAIL		***Ex vivo*** EVs directly triggered caspase activation and apoptosis in tumour cells: EVs cytotoxicity against B16 melanoma (maximal at 48 h): EVs from immature DCs (iEVs) – 10-30% of specific lysis. EVs from mature DCs (mEVs) – 20-60% of specific lysis. mEVs cytotoxicity against KLN205 lung squamous carcinoma – 3-35% of specific lysis. mEVs cytotoxicity against MC38 colon adenocarcinoma – 10-22% of specific lysis. EVs activated NK cells and stimulated them to secrete IFN-γ upon the interaction of transmembrane TNF on EVs with TNF receptors on the surface of NK cells.	Munich et al., 2012
Murine BM-DCs	Sequential centrifugation		TLR-ligands: LPS (TLR4 ligand) Pam_3_ synthetic (TLR1/2 ligand)	Direct	***Model 1.*** **Incubation of LPS-iEVs or LPS-mEVs with DCs and aNK cells** ***in vitro*** (1) LPS-EVs activated bystander DCs by ↑ expression of transmembrane TNF (tmTNF) and soluble TNF. (2) enhanced iDCs/aNK crosstalk – interaction between tmTNF-DCs with TNFR2-aNK resulted in ↑ IFN-γ by aNK. Pam_3_-EVs had similar effects. ***Model 2.*** **Incubation of Pam** **_3_** **-mEVs with splenocytes** ***in vitro*** Pam_3_-mEVs ↑ iDCs/NK crosstalk (↑ secretion of IFN-γ by NK).	Sobo-Vujanovic et al., 2014
Murine BM-DCs matured with either poly(I:C) (TLR-3L), LPS (TLR-4L) or CpG-B (TLR-9L)	Sequential centrifugation		OVA Antigens from HOCl-oxidized necrotic B16-F10 cells	Indirect	Poly(I:C) is particularly favorable TLR agonist for DC maturation during antigen loading and EVs production for cancer immunotherapy. ***In vivo*** **OT-I adoptive transfer model** Poly(I:C)-OVA-EVs (IV or ID injected in mice) stimulated proliferation of OVA-specific CD8^+^ and CD4^+^ T cells, activated Th1 immune response. ***In vivo*** **B16-F10 melanoma model** Therapeutic settings Vaccines (ID injected in mice): Poly(I:C)-B16-EVs LPS-B16-EVs CpG-B-B16-EVs All EVs formulations were able to ↓ growth of B16-F10 tumours. Poly(I:C)-B16-EVs were the most efficient – induced robust activation of melanoma-specific CD8^+^ T cells in tumour-draining lymph nodes, spleen and tumour mass; activated recruitment of NK and NK-T cells to the tumour site; significantly ↓ tumour growth and ↑ survival of diseased animals.	Damo et al., 2015
Murine spleen DCs	Total exosome isolation kit (Invitrogen)				Heat stressed and high CO_2_-treated EVs (HS-CO_2_-EVs). ***Ex vivo*** HS-CO_2_-EVs inhibited AGS gastric cancer cell prolipheration (55% inhibition), induced tumour apoptosis (28% tumour apoptotic cells). ***In vivo*** **AGS human gastric cancer xenograft model** HS-CO_2_-EVs inhibited AGS tumour growth in 1.54 times as compared with control group in nude mice.	Wang et al., 2015
C. EVs-based anti-tumour immunotherapy in clinical trials.						
Tumour type	Phase	Number of patients	EV origin; isolation method	Carried/loaded molecules	Outcome	References
Non-small cell lung cancer	Phase 2	22	Monocyte-derived DCs; Ultrafiltration / diafiltration → sucrose ultracentrifugation	IFN-γ MHC I-peptides: MAGE-A1, MAGE-A3, NY-ESO-1, Melan-A/MART-1 MHC II peptides: MAGE-A3-DP04, EBV.	**Immunologic results** ↑ NKp30-dependent NK cell functions. **Clinical results** Progression-free survival – 50% of patients at 4 months after chemotherapy cessation. 1/22 – grade three hepatotoxicity. 7/22 – disease stabilization of > 4 months. Median time to progression – 2.2 months. Median overall survival – 15 months.	Besse et al., 2016
Colorectal cancer	Phase 1	40	Tumour cells from ascites; sucrose/D_2_O density gradient ultracentrifugation		Vaccination of patients with EVs alone or in combination with GM-CSF were safe and well tolerated. EVs+GM-CSF induced beneficial tumour-specific antitumour CTL response.	Dai et al., 2008
Melanoma IIIB/IV stage	Phase 1	15	Monocyte-derived DCs; Ultrafiltration, sucrose ultracentrifugation	MAGE3 peptides	There was no grade II toxicity → maximal tolerated dose was not achieved. **Immunological results** MAGE3-specific CD4^+^ and CD8^+^ T cell responses were not detected. **Clinical results** 1/15 – partial response (according to RECIST criteria). In skin and lymph nodes: 1/15 – minor response. 2/15 – stable response. 1/15 – mixed response.	Escudier et al., 2005
Advanced melanoma	Phase 1	15	Monocyte-derived DCs; Ultrafiltration / diafiltration, Sucrose / D_2_O cushion ultracentrifugation	MAGE.A1 and MAGE3.DP04	**Immunological results** 7/14 – restored the number and NKG2D-dependent function of NK cells	Viaud et al., 2009
Non-small cell lung cancer	Phase 1	9	Monocyte-derived DCs;	MAGE peptides	**Immunological results** No major toxicity. 3/9 – MAGE-specific T cell responses; 2/9 – ↑ increased NK cell lysis	Morse et al., 2005

aNK, activated NK (splenocytes activated with IL-2 for 6 days); CRCL, chaperone-rich cell lysate; CTLs, cytotoxic T-lymphocytes; DCs, dendritic cells; EVs, extracellular vesicles; GM-CSF, granulocyte-macrophage colony-stimulating factor; HS, heat stressed; ID, intradermal; iDCs, immature DCs; iEVs, extracellular vesicles from immature DCs; IFN, interferon; IL, interleukin; IT, intratumoural; lEVs, large extracellular vesicles; LPS, lipopolysaccharide; mEVs, extracellular vesicles from mature DCs; MVs, microvesicles; NK, natural killer; OVA, ovalbumin; PBMC, peripheral blood mononuclear cells; PT, peritumoural; SC, subcutaneous; sEVs, small extracellular vesicles; TGF, transforming growth factor; Th, T-helper cells; tmTNF, transmembrane tumour necrosis factor; TNF, tumour necrosis factor; Tregs, T-regulatory cells.

To increase the enrichment and purity of isolated EVs, centrifugation using a sucrose density gradient or sucrose cushion, in addition to ultrafiltration, is used in combination with ultracentrifugation or alone (Dai et al., 2008; Bu et al., 2011; Besse et al., 2016; Diamond et al., 2018; Guo et al., 2018). Sucrose-based density gradient ultracentrifugation of EVs allows isolation of the pure fraction of EVs due to their specific buoyant density—exosomes float in a sucrose gradient of 1.13 to 1.19 g/mL (Théry et al., 2002). The ultrafiltration method sequentially removes larger particles from samples, with a final ultrafiltration step using a 100-kDa MWCO filter, such as Centricon Plus-70, Centriplus, or Amicon Ultra (Millipore). This method is rapid and technically easy but allows isolation of EVs with high purity. Furthermore, a combination of filtration and sucrose-based density gradient centrifugation has made it possible to efficiently isolate high purity EVs in sufficient amounts for clinical trials (Escudier et al., 2005; Morse et al., 2005).

Easy-to-use commercial kits, such as ExoQuick (System biosciences) (Rekker et al., 2014) or the Total Exosome Isolation Kit (Invitrogen) (Wang et al., 2015), are rarely used to isolate DC-derived EVs. The principle of such kits is salting out EVs from samples by the addition of water-excluding polymers, such as polyethylene glycol (PEG), which occupy water molecules and force less soluble components, including EVs, out of the solution (Li et al., 2017). Following overnight incubation at 4°C, precipitated EVs are isolated by short centrifugation steps at low speed (up to 10,000*g*). The advantages of commercial kits are less time-consuming method, possibility to isolate EVs from small volumes of medium, no need for special expensive instruments, such as an ultracentrifuge, and no technical challenges (Helwa et al., 2017). However, the main drawback of this method is the isolation of non-vesicular particles together with EVs (Yamada et al., 2012), which is the reason why this method is less frequently used.

Other methods of EV isolation, such as immunoaffinity capture-based and microfluidics-based isolation techniques, are more expensive and technically complicated, and in practice are not used for the isolation of DC-derived or DC-targeted MVs. Therefore, these approaches are not considered in the present review. The principles of the different EV isolation techniques are described in detail in other reviews (Li et al., 2017; Pariset et al., 2017).

## Methods of EV Loading With Therapeutic Molecules

For proper activation of an anti-tumor immune response, the antigen-presenting cells (APC), in particular DCs, should convey three signals to T cells: (1) presentation of tumor antigens in complexes with MHC class I and II molecules on the surface of DCs to T-cell receptors (2) signal transmission via interaction of co-stimulatory and adhesion molecules expressed on the surface of DCs to their receptors on T cells; and (3) production of T-cell stimulatory cytokines by DCs (Kapsenberg, 2003).

It is known that DC-derived EVs carry all the molecules required to activate anti-tumor T cell-mediated immune responses (Seo et al., 2018). On their surface, DC-derived EVs contain functionally active complexes containing tumor antigens and MHC molecules class I and II, as well as co-stimulatory and adhesion molecules (Morelli et al., 2004; Chaput et al., 2006). Furthermore, it has been shown that EVs are able of carrying cytokines (Gulinelli et al., 2012). Therefore, DC-derived EVs are assumed to interact with T lymphocytes and directly trigger anti-tumor immune responses that has been proven by many experimental data (Wang et al., 2013; Lu et al., 2017; Chen et al., 2018 etc.) (see **Table 2B**).

With respect to tumor cell-derived DC-targeted EVs, these vesicles may transfer tumor antigenic peptides and immunostimulatory molecules to DCs, resulting in the induction of DCs exhibiting high immunogenicity (Pitt et al., 2014).

Generally, for proper activation of immune responses, in addition to tumor-associated antigens and immunostimulatory molecules, DC-derived and DC-targeted EVs should carry tumor-targeted molecules. EVs can also carry therapeutic NAs, such as small non-coding regulatory RNA (siRNA, miRNA) for the targeting of certain genes in recipient cells, thus enhancing immunogenicity or inhibiting negative signals (Jiang et al., 2017). The efficient loading of EVs with therapeutic molecules is the most important step in the preparation of EV-based anti-tumor vaccines. Therapeutic molecules can be delivered to EVs indirectly by the loading of EV-secreting cells followed by the isolation of EVs or directly by the loading of preliminarily isolated EVs (Batrakova and Kim, 2015). Summarizing the investigations performed over recent decades, in the next part of the present review we will discuss the molecules used for the loading of anti-tumor EVs and provide an overview of the methods used for the indirect or direct loading of EVs with therapeutic molecules.

### Loading of EVs With NAs

NA-based therapeutics is a promising tool for the modification of immune cell properties and treatment of a variety of human diseases. Plasmid DNA and mRNA encoding tumor antigens, immunostimulatory molecules, EV-targeted proteins, and regulatory short non-coding RNA (miRNA, siRNA), can be successfully used to launch or enhance the anti-tumor potential of EV-based vaccines (Van den Boorn et al., 2013). The main problems with NA application are instability of the naked NA in the presence of nucleases as well as non-targeted delivery. As a result, various types of chemical modifications of NAs and nanocarriers have been developed to ensure the stability of NAs and to provide their targeted delivery to both cells and EVs.

Overall, only two strategies are used to load EVs with NAs: (1) indirect loading – preliminary transfection/transduction/electroporation of EV-secreting cells with NA followed by EVs isolation or (2) direct loading of preliminarily isolated EVs with the NA (Johnsen et al., 2014).

#### Indirect Loading of EVs With NA

Indirect loading of EVs is achieved by preliminary delivery of NA to EV-secreting cells by transfection, transduction, or electroporation. Subsequently, NA may be sorted into MVBs and secreted by exosomes (in the case of regulatory small non-coding RNA, such as siRNA or miRNA) or NA can be translated to peptides or proteins (tumor peptides, chimeric proteins, targeting molecules) that can be carried by EV to immune or tumor cells.

This approach has been well adapted for miRNA and siRNA loading. Overexpression of miRNA in EV-secreting cells is typically realized by transfection of cells with plasmid DNA or viral vectors encoding miRNA (Katakowski et al., 2013; Wang et al., 2016), while siRNA, in addition to the above-mentioned methods, can be delivered to cells in the form of siRNA duplexes. Recent reports have revealed that the use of siRNA duplexes is preferred (Zhang et al., 2014b) and allows packaging of approximately 0.001 to 0.14 pmol siRNA into 1 μg EVs (Zhang et al., 2014b; Liu et al., 2015). It was revealed that indirect loading of murine leukemia-derived EVs with *TGF-β1* shRNA resulted in robust maturation of DCs, activation Th1 immune response and pronounced inhibition of tumor growth *in vivo* (Huang et al., 2017).

The loading of high-molecular weight NAs, such as plasmid DNA and mRNA, into EVs is associated with additional difficulties, such as low loading efficiency due to the size of NAs and the possibility of losing functional activity of NAs. Thus, in the case of high-molecular weight NAs, the common strategy for the modification of EVs is preliminary transfection/transduction/electroporation of EV-secreted cells.

Using the indirect NA delivery technique, DC-derived or tumor-derived DC-targeted EVs can be modified with different proteins, such as tumor-associated antigens or DC-activating molecules, respectively, by preliminary transfection/transduction of EV-producing cells with plasmid DNA/RNA/viral vectors encoding these proteins. Concerning the modification of DC-derived EVs with tumor antigens, DCs can be transduced with viral vectors encoding full-length tumor-associated proteins (Wang et al., 2013; Lu et al., 2017) or transfected with tumor RNA encoding a pool of tumor-associated proteins and peptides (Gehrmann et al., 2013). Following transfection/transduction of cells, intracellular processing of proteins occurs, and complexes of tumor peptides with MHC molecules class I and II are formed and exposed on the surface of EVs. Such DC-derived EVs carrying tumor-associated peptides in complex with MHC molecules are able to directly activate highly efficient T-cell anti-tumor immune responses both *in vitro* and *in vivo* (Gehrmann et al., 2013; Wang et al., 2013; Lu et al., 2017).

To place a particular peptide or protein on the surface of EVs, indirect loading of EV-producing cells with NA encoding chimeric proteins consisted of EV surface protein (lactadherin, Lamp2b, etc.) fused with protein or another molecule of interest (tumor antigen, immunostimulatory molecules as well as DC- or tumor-targeted molecules) can be applied.

For example, lactadherin, exosome-specific anchor expressed on the surface of EVs, can be used to modify EVs. EV-secreting murine melanoma cells were transfected with plasmid DNA encoding lactadherin fused to streptavidin; the EVs produced by these cells were shown to express streptavidin on their surface and could be modified with either biotinylated CpG DNA (Morishita et al., 2016) or GALA peptide (Morishita et al., 2017) for activation of DC maturation or enhancement of EV cargo release into the cytosol of recipient cells, respectively. Moreover, lactadherin was also fused to the tumor-associated antigens, CEA and HER2, to modify EVs produced by HER2^+^ breast carcinoma cells. Adenoviral vectors encoding lactadherin-CEA or lactadherin-HER2 fusion proteins were used to transduce EV-secreting cells. Enhanced expression of tumor antigens on the surface of EVs resulted in significant activation of antigen-specific anti-tumor immune responses in animal models of breast tumors (Hartman et al., 2011).

A mutant form of the HIV-1 Net protein (Net^mut^), that has been shown to have extraordinarily high levels of accumulation in exosomes (Lattanzi and Federico, 2012), can be used as anchors to modify EVs. Plasmid DNA encoding Net^mut^ fused to the tumor antigens, HER2 or MART-1, was employed to indirectly modify human muscle cell-derived EVs. Modified EVs were demonstrated to possess great anti-tumor potential against breast cancer and lymphoblastoma *in vitro* and *in vivo* (Anticoli et al., 2018).

EVs possess the natural property of transferring their contents to target cells and tissues; however, in their unmodified form, EVs are known to accumulate mainly in the liver, kidneys, intestine, lungs, and spleen of laboratory animals (Wiklander et al., 2015). Accordingly, many studies have been conducted with the aim of targeting EV/NA complexes to organs and tissues of interest (Alvarez-Erviti et al., 2011; Tian et al., 2014; Liu et al., 2015; Bellavia et al., 2017). Modification of EVs with DC- or tumor cell-targeted molecules can allow an increase in the delivery efficiency of miRNA and siRNA to DCs or tumor cells and enhance the biological effect of therapeutic NA. This approach can be performed by transfection or transduction of EV-secreting cells with plasmid DNA or viral vectors, respectively, encoding EV surface proteins fused to DC-/tumor-targeted or immunostimulatory molecules.

For instance, it has been demonstrated that the Lamp2b protein expressed on the surface of EVs can be modified by fusion with the RVG-peptide (neuron-specific rabies viral glycoprotein that binds to acetylcholine receptors) to target DC-derived EVs to neuronal cells *in vitro* and to brain cells *in vivo* (Alvarez-Erviti et al., 2011; Liu et al., 2015). Application of α-bungarotoxin (inhibitor of acetylcholine receptors) has been shown to result in the loss of function of RVG-EVs with respect to the ability to transfer BACE1 siRNA to Neuro2A cells (Alvarez-Erviti et al., 2011). In another study, DC-derived EVs were modified with Lamp2b by fusion with the iRGD peptide targeted to αv-integrin-positive breast cancer cells. It was demonstrated that these iRGD-modified EVs more effectively penetrated into MDA-MB-231 cells than unmodified EVs. Moreover, intravenous injection of iRGD-EVs into tumor-bearing mice has been reported to result in the rapid accumulation of reprogrammed EVs in tumors (Tian et al., 2014).

Modification of DC-targeted tumor-derived EVs involves preliminary transfection of EV-producing cells with plasmid DNA encoding DC-targeted/-activating molecules, for example CD40L (Wang et al., 2014). It was shown that EVs derived from CD40L-modified Lewis lung carcinoma cells were highly immunogenic towards DCs *in vitro* and extremely efficient in a protective and therapeutic scheme of treatment for murine lung carcinoma *in vivo* (Wang et al., 2014).

It appears that reprogrammed EVs have a similar transfection efficiency to commercial transfection reagents. It has been reported that RVG-EVs have a transfection efficiency comparable with commercial TransIT LT1 transfection reagent (Mirus Bio). In this work, DC-derived RVG-modified EVs transferred three types of α-Syn siRNA to a human SH-SY5Y neuroblastoma cell line expressing mouse α-Syn-HA. The level of α-Syn mRNA/protein reduction was similar, with a slight advantage for RVG-EVs over TransIT LT1-mediated delivery (Cooper et al., 2014). Moreover, a similar result has been demonstrated previously with RVG-modified murine DC-derived EVs (Alvarez-Erviti et al., 2011). The delivery of *GAPDH* and *cyclophilin B* siRNA was performed by reprogrammed RVG-EVs, and the level of gene silencing was comparable with that obtained following transfection with Lipofectamine 2000 (Thermo Fisher Scientific) transfection reagent (Alvarez-Erviti et al., 2011).

#### Direct Loading of EVs With NA

In addition to the indirect modification of EVs by preliminary cell transfection, another approach is the direct modification of EVs with DC-/tumor-targeted molecules.

Electroporation is often used for direct loading of EVs with low-molecular weight NAs, with an efficiency of approximately 3% to 24% transfected EVs (Alvarez-Erviti et al., 2011; Wang et al., 2017; Usman et al., 2018). This technique has been applied to EVs of different origins including DCs (Alvarez-Erviti et al., 2011; Wang et al., 2017). Electroporation is best suited to the direct loading of EVs with small non-coding regulatory RNAs and antisense nucleotides (ASOs). For instance, miRNA-155 was efficiently directly loaded into EVs derived from murine colon carcinoma cells by using electroporation (Asadirad et al., 2019). miRNA-155-EVs was shown to significantly enhance maturation of DCs and stimulate their immunostimulatory functions (Asadirad et al., 2019). One of the main problems with electroporation is the aggregation of EVs, which result in distortion of the true efficiency of NA incorporation into EVs (Kooijmans et al., 2013; Usman et al., 2018).

In addition, small non-coding RNA can be directly delivered to EVs in the form of conjugates with hydrophobic molecules, such as cholesterol. This approach is prospective, since it allows loading of a large number of NA molecules into EVs in the absence of any transfection reagents and additional manipulations (Didiot et al., 2016). Nevertheless, the functional activity of cholesterol-conjugated siRNA can be lost in recipient cells (Stremersch et al., 2016). Indeed, melanoma B16 or monocyte/DC-derived EVs loaded with cholesterol-conjugated siRNA (chol-siRNA) were used to silence *CD45* and e*GFP* genes in JAWSII monocytes and H1299 non-small cell lung carcinoma cells, respectively. Highly efficient loading of EVs with chol-siRNA was shown; specifically, 15 μg EVs (∼6.6 × 10^10^ vesicles) were able to bind 80% of the 10 pmol chol-siRNA, corresponding to approximately 73 chol-siRNA molecules per vesicle (Stremersch et al., 2016). EVs loaded with chol-siRNA were demonstrated to efficiently penetrate the target cells; however, no downregulation of gene expression in either tested cell line was observed (Stremersch et al., 2016). In another study, it was reported that approximately 74% of chol-siRNA was loaded into Neuro2A- or DC-derived EVs by using electroporation, and subsequently recommended to use roughly 15 molecules of chol-siRNA per vesicle to achieve this efficiency. In this case, EVs loaded with chol-siRNA were able to transport functionally active siRNA to target cells and downregulate target genes in a concentration-dependent manner (O’Loughlin et al., 2017). The loss of functional activity of chol-siRNA delivered by EVs observed in the Stremersch’s investigation was possibly associated with high affinity between EVs and chol-siRNA, and hence, inability to release siRNA into the cytosol for biological action to occur. Furthermore, the structure and length of the linker between siRNA and cholesterol molecules are of great importance to ensure the biological activity of chol-siRNA conjugates (Petrova et al., 2012).

A technique for direct targeting EVs to tumor cells is the labeling of EVs with aptamers. This technique can be conveniently used for artificial EV-mimics derived from cell membranes. For example, the tumor-targeted anti-nucleolin aptamer, AS1411, conjugated to cholesterol-poly(ethylene glycol) was used to modify the membrane of murine DCs, which were further processed by extrusion to generate artificial mimics of natural EVs that significantly eradicated tumors in MDA-MB-231 breast tumor xenograft model (Wan et al., 2018). In another study, murine DC-derived EVs were also modified by AS1411. The binding efficiency of AS1411-modified EVs to breast cancer cells was roughly fourfold greater in comparison with unmodified EVs (Wang et al., 2017). Intravenous injection of miR-let-7–loaded AS1411-modified EVs or control EVs to breast tumor-bearing mice resulted in much higher intratumoral accumulation of the AS1411-modified EVs in comparison with unmodified EVs that resulted in more significant retardation of MDA-MB-231 tumor growth (Wang et al., 2017).

At the end of this section, we briefly consider some other techniques for NA loading into EVs that were recently used to prepare anti-tumor EVs. In some studies, transfection reagents, for example Lipofectamine 2000 (Thermo Fisher Scientific), were used for the direct loading of EVs with NA (Wahlgren et al., 2012; Shtam et al., 2013; George et al., 2018). The main problems of using chemical transfectants to directly load EVs are formation of surface conglomerates of NA/transfectant complexes with EVs that can drastically alter the original vesicle composition (Wahlgren et al., 2012). In other studies, CaCl_2_-associated transfection of isolated EVs combined with heat shock at 42°C has been reported (Zhang et al., 2017; Zhang et al., 2018). This approach allows the loading of approximately 200 copies of siRNA per EV. Sonication has also been used for NA loading into EVs, showing a significant decrease in siRNA aggregation in comparison with electroporation (Lamichhane et al., 2016). Moreover, the promising approach of using freezing/thawing and extrusion methods, in addition to a permeabilization technique with saponin, has been demonstrated for the efficient loading of EVs (Haney et al., 2015).

### Direct and Indirect Modification of EVs With Proteins and Peptides

To use EVs as immunotherapeutic cell-free vaccines, DC-derived EVs can be modified not only with NAs but also with tumor-associated proteins or peptides. Similar to NA delivery into EVs, there are two techniques for loading EVs with proteins/peptides: indirect loading of EV-secreting cells and direct loading of EVs.

This technique allows to prepare EVs that are able to activate tumor antigen-specific immune responses. Both indirect and direct loading of EVs with proteins/peptides leads to the presentation of tumor-associated peptides in complexes with MHC class I and II molecules on DC-derived EVs and promotes the interaction of these EVs with T-lymphocytes or NK cells and subsequent activation of anti-tumor immunity.

Indirect loading of DC-derived EVs was demonstrated with the tumor-specific proteins or peptides MAGE3 (Viaud et al., 2009) and HPV early antigen 7 (Chen et al., 2018), model tumor antigens, such as OVA and OVA-derived peptide SIINFEKL (Gehrmann et al., 2013; Naslund et al., 2013; Yao et al., 2013; Damo et al., 2015; Wahlund et al., 2017), tumor lysates (Guan et al., 2014; Bu et al., 2015), and even HOCl-oxidized B16 melanoma cells containing both proteins and NAs (Damo et al., 2015). Immature DCs possess the intrinsic ability to capture proteins and peptides from surrounding fluids and tissues; therefore, there is no need to use special reagents or techniques for loading (Savina and Amigorena, 2007). Captured proteins and peptides are processed and tumor antigens in complex with MHC class I and II molecules are subsequently exposed on the surface of DCs, and as a consequence, on DC-derived EVs (Pitt et al., 2014; Markov et al., 2016). The presence of tumor peptides on the surface of EVs is proven in most cases by pentamer staining, ELISA, or western blotting assays. It should be mentioned that the maturation status of DCs loaded with proteins/peptides is essential for expression of costimulatory and adhesion molecules on the surface of DC-derived EVs, which are needed for the induction of an efficient anti-tumor immune response (Lutz and Schuler, 2002). Hence, the choice of suitable maturation stimuli for the treatment of DCs is of great importance. Different compounds have been used to stimulate the maturation of DCs, such as LPS, poly(I:C), CpG-oligonucleotide, and TNF-α. It has been shown that mature DC-derived EVs indirectly loaded with tumor antigens activate efficient anti-tumor immune responses *in vitro* and *in vivo* (Naslund et al., 2013; Bu et al., 2015; Damo et al., 2015; Wahlund et al., 2017; Chen et al., 2018) (see DC-Derived EVs and **Table 2B**).

The technique of direct loading of EVs with tumor antigenic peptides was developed by Hsu and co-authors (Hsu et al., 2003). Direct binding of peptides to MHC I and II molecules on the surface of DC-derived EVs was performed under mildly acidic loading conditions. It was demonstrated that MHC class I molecules on EVs can be directly loaded with peptides, such as HLA-A2–restricted MART1 tumor peptide, at much greater levels than indirect loading, requiring a 100- to 1000-fold lower peptide concentration. Moreover, it was demonstrated that MHC I molecules efficiently bind tumor peptides at pH 4.2 in the presence of β_2_-microglobulin (β_2_m); in the absence of β_2_m, MHC I molecules also efficiently bind tumor peptides under less acidic conditions (pH 5.2). Although the efficiency of peptide binding at pH 5.2 in the absence of β_2_m is approximately 50% of that observed at pH 4.2 in the presence of β_2_m, the biological activity of DC-derived EVs prepared in accordance with both methods is comparable (Hsu et al., 2003). MHC II molecules that are expressed in high density on the surface of DC-derived EVs also efficiently bind tumor peptides under the same conditions. Obtained complexes of MHC I or MHC II with tumor peptides maintain complete functional activity; thus, the technique of direct loading of DC-derived EVs with peptides consists not of peptide delivery inside EVs but the binding of peptides with MHC class I and II molecules on the surface of EVs. In this form, EVs can stimulate both CD8^+^ cytotoxic T-lymphocytes and CD4^+^ T-helper cells, which are crucial for triggering an efficient anti-tumor immune response (Hsu et al., 2003). More recently, this technique was used to load DC-derived EVs with the tumor-associated MAGE3 peptide to treat advanced melanoma in clinical trial (Viaud et al., 2009). Vaccination of patients with obtained EVs resulted in activation of NK cells (Viaud et al., 2009).

## Anti-Tumor Potential of DC-Derived and DC-Targeted Evs *in Vitro* and *in Vivo*

### Tumor Cell-Derived DC-Targeted EVs

The anti-tumor potential of DC vaccines primarily depends on the efficiency of DC loading with tumor antigens. Tumor-derived EVs are known to be natural transport vectors that carry a large variety of tumor antigens, and thus can serve as vesicles for the direct delivery of tumor antigens to DCs. Current success in application of tumor-derived DC-targeted EVs to treat tumors in different murine models *in vivo* is summarized in **Table 2A**.

The ability of tumor cell-derived EVs to induce an anti-tumor immune response depending on their size has been investigated, and large-sized tumor cell-derived EVs (MVs, diameter 100−1000 nm) were shown to be more immunogenic than small-sized EVs (exosomes, diameter 60−100 nm). Treatment of tumor-bearing mice with large- and small-sized EVs resulted in the inhibition of tumor growth, with 50% and 12.5% of mice, respectively, remaining tumor-free. Furthermore, treatment of mice with tumor cell-derived large-sized EVs resulted in the activation of anti-tumor cytotoxic T-lymphocytes (CTLs) that were 1.5-fold to 2-fold more efficient in comparison with CTLs activated by treatment with small-sized EVs (Zhang et al., 2014a). It should be mentioned that despite the successful application of cell-free vaccines based on DC-targeted tumor-derived EVs, DCs pre-loaded with tumor-derived EVs inhibited tumor growth significantly more efficiently in comparison with EV-based vaccines in all tested tumor models (Zhang et al., 2014a). This can be explained by the fact that tumor-derived EVs are not specifically targeted to DCs, and following subcutaneous inoculation in mice, interact with cells of different origins, not only with DCs, thus losing their antitumor potential.

Indeed, it was revealed that DCs loaded with EVs produced by murine L1210 leukemia cells activate a more efficient anti-tumor immune response in both prophylactic and therapeutic regimens in comparison with cell-free EV-based vaccines. In a model of L1210 murine leukemia, it was shown that subcutaneous injection of DC/L1210-EVs at a dose of 4×10^6^ cells per mouse results in complete eradication of tumors in a therapeutic setting and protection of all mice in a prophylactic setting, whereas vaccines based on tumor-derived EVs are not as efficient (Yao et al., 2014).

The next question is whether DCs loaded with tumor-derived EVs are more immunogenic than classic DC-based vaccines loaded with tumor proteins or NA encoding tumor antigens. On the basis of the facts that tumor vesicles contain large amounts of different tumor antigens (Wolfers et al., 2001) and have a natural ability to efficiently deliver cargo to the cells (Ha et al., 2016), it appears that the use of tumor-derived EVs as a source of tumor antigens to load DCs is superior to commonly used sources of tumor antigens (tumor proteins, peptides, or NAs). Indeed, it has been shown that human DCs loaded with glioma cell-derived EVs activate anti-tumor CTLs *ex vivo* that kill glioma cells two-fold more efficiently in comparison with CTLs primed with tumor lysate-pulsed DCs (Bu et al., 2011). Using a mouse AB1 malignant mesothelioma model, it was revealed that treatment of tumor-bearing mice with DCs loaded with either tumor cell-derived EVs or tumor lysate results in infiltration of CD4^+^ T cells, CD8^+^ T cells, and DCs into tumor tissues. However, in the case of EV-loaded DCs, a higher increase in overall and median survival of experimental animals was observed as compared with lysate-pulsed DCs: the overall survival (on the 52^nd^ day of tumor development) was 0%, 16.7%, and 33.3%; and the median survival was 13, 18.5, and 29.5 days for mice treated with PBS, lysate-pulsed DCs, and EV-loaded DCs, respectively (Mahaweni et al., 2013). Greater immunogenicity of tumor cell-derived EV-loaded DCs has also been demonstrated. Treatment of WEHI3B myeloid leukemia-bearing mice with such EV-loaded DCs resulted in a more significant retardation of tumor growth and survival in animals as compared with treatment with lysate-pulsed DCs (Gu et al., 2015). The efficiency of DC-based vaccines loaded with tumor cell-derived EVs was shown to be almost equal to that of protein-pulsed DCs. Thus, DCs loaded with EVs derived from B16-F10-OVA mouse melanoma cells were shown to activate anti-tumor and anti-metastatic immune responses *in vivo* with slightly less intensity than that evoked by OVA-loaded DCs: tumor occurrence was observed in one of eight mice and zero of eight mice, and the mean number of metastases was 5 and 0, respectively (Yao et al., 2013). Such high efficiency of protein-pulsed DCs is suggested to be associated with the model tumor antigen OVA, which is abundantly expressed in tumor cells and does not reflect the actual expression of tumor-associated antigens. When tumor-associated antigens were used to load DCs or DC-derived EVs, higher immunogenicity of EV-loaded DCs over oncoprotein-loaded DCs could be explained by prolonged presentation time and enhanced recovery of EVs in comparison with tumor proteins (Gu et al., 2015)

To enhance the immunogenicity of tumor-derived EVs, EV-secreting tumor cells or EVs themselves undergo modifications with different molecules or exposure to radiation. Tumor cell-derived EVs were shown to be indirectly (see Loading of EVs with NA) modified with the DC-targeted/-activating molecule CD40L (Wang et al., 2014), the Rab27a molecule that takes part in exosome secretion (Li et al., 2013), the shRNA silencing immunosuppressive TGF-β1 (Huang et al., 2017), or degraded cytosolic DNA (Diamond et al., 2018), as well as the pH-sensitive GALA-peptide that contributes to the endosomal release of EVs into the cytosol of DCs (Morishita et al., 2017) or the immunostimulatory CpG DNA (Morishita et al., 2016). All modified tumor cell-derived EVs mentioned above showed high immunostimulatory activity that exceeded the activity of unmodified EVs. Thus, it was demonstrated that DC-targeted tumor cell-derived EVs can be successfully used for the loading of DCs, and as such, tumor EV-loaded DC vaccines had higher anti-tumor efficiency in comparison with classic DC vaccines.

Nevertheless, it should be mentioned that it is necessary to carefully test tumor-derived EVs during preparation of anti-tumor vaccines, since tumor-derived EVs despite immunotherapeutic potential possess immunosuppressive properties that can lead to inhibition of immune cell functions and as a result to escape of tumor from immunosurveillance and formation of metastatic niche. A short review of immunosuppressive action of tumor-derived EVs on immune cells is presented below.

#### Immunosuppressive Action of Tumor Cell-Derived DC-Targeted EVs

As indicated above, tumor-derived EVs were successfully applied as efficient source of tumor antigens to load DCs that eventually activated robust anti-tumor immune response in different murine tumor models (see Tumor Cell-Derived DC-Targeted EVs and **Table 2**). Nevertheless, immunotherapeutic potential of tumor-derived EVs is confused and controversial. It was demonstrated that tumor-derived EVs along with immune stimulatory action could also deliver tolerogenic signals to immune cells (Whiteside, 2016b).

The immunoinhibitory effects of tumor-derived EVs on immune cells can be direct (when signals or cargo delivered by EVs inhibit the targeted cell functions) or indirect (when EVs reprogram the differentiation program of targeted cells, which then suppress functions of other cells) (Whiteside, 2016b). Indeed, on the one hand, tumor-derived EVs carry FasL and TRAIL molecules and can directly induce apoptosis of DCs (Peng et al., 2011; Ning et al., 2018) or effector CD4^+^ and CD8^+^ T-lymphocytes (Whiteside, 2016a). On the other hand, tumor-derived EVs can block differentiation of myeloid progenitor cells into CD11^+^ DCs and direct them to differentiate into myeloid-derived suppressor cells (Xiang et al., 2009; Ning et al., 2018) or inhibit maturation and migration of DCs (Yang et al., 2011; Ning et al., 2018) that results in immunosuppression and favors tumor immune escape.

It was demonstrated that HSP72 and HSP105 expressed on the surface of tumor-derived EVs promoted DCs to produce high levels of pro-inflammatory cytokines (IL-6, PGE2, IL-1β, TNF-α) in a TLR2- and TLR-4–dependent manner (Shen et al., 2017). High levels of IL6 dramatically promoted tumor invasion and metastasis of murine melanoma by upregulation of transcription activity of STAT3 and production of STAT3-dependent MMP9 in tumor cells. It should be mentioned that depletion of IL-6 converted tumor-derived EVs from tumor promoters to inhibitors of tumor metastasis *in vivo* (Shen et al., 2017).

Human pancreatic cancer cell-derived EVs were shown to carry miR203 that inhibited expression of TLR4 and blocked secretion of IL-12 and TNF-α immunostimulatory cytokines in human DCs (Zhou et al., 2014).

Furthermore, tumor-derived EVs could drive differentiation and expansion of Treg cells (Huang et al., 2013; Whiteside, 2016a; Ning et al., 2018). Tumor cell-derived EVs induced the conversion of human conventional CD4^+^CD25^–^ T cells to CD4^+^CD25^hi^FOXP3^+^ Treg cells in a TGF-β1–dependent manner (Wieckowski et al., 2009). Treg cells coincubated with tumor EVs were shown to upregulate the expression of FasL, TGF-β, IL-10, CTLA4, granzyme B, and perforin and exhibited enhanced suppressor functions (Szajnik et al., 2010).

NK cells being highly important in anti-tumor immunity due to induction an antigen-independent immune response against malignant cells (Fang et al., 2017) could be also inhibited by tumor cell-derived EVs (Whiteside, 2016b). It was demonstrated that NKG2D ligands (MICA, MICB, and ULBP) carried by tumor-derived EVs bound to the inhibitory receptor, NKG2D, on the surface of NK cells, with delivering of inhibitory signals and blocking of anti-tumor cytotoxicity of NK cells (Clayton et al., 2008; Lundholm et al., 2014). Another mechanism of tumor EV-mediated inhibition of NK functions was attributed to the presence of TGF-β1 in EVs cargo, a cytokine suppressing cytotoxicity of NK cells (Szczepanski et al., 2011).

It can be assumed that the choice of the immunostimulatory or immunosuppressive effects of the tumor cell-derived EVs depends on many factors: isolation methods of EVs from tumor cells, DC loading conditions, used immunostimulating molecules, the stage of the tumor process, and so on. In any case, preparation of antitumor vaccines on the basis of tumor cell-derived EVs should be associated with comprehensive and thorough investigation of their all biologic effects on immune system. On the other hand, the immunosuppressive properties of the tumor cell-derived EVs can be successfully applied to treat autoimmune diseases or to reduce graft-to-host immune reactions.

### DC-Derived EVs

DC-derived EVs carry all the molecules needed for activation of a T-cell immune response and can act alone as cell-free anti-tumor vaccines. To efficiently activate anti-tumor immune responses by DC-derived EVs, the proper choices of tumor antigens for the loading of EV-producing DCs and factors stimulating the maturation of DCs, are of great importance. Significant success in the treatment of tumors by DC-derived EVs has been achieved in murine tumor models *in vivo* and human cells *ex vivo* (see **Table 2B**).

The anti-tumor potential of cell-free vaccines based on murine DC-derived EVs indirectly loaded with α-fetoprotein (AFP) has been evaluated (Lu et al., 2017). It was demonstrated that AFP-EVs activate an efficient antigen-specific immune response that causes significant retardation of tumor growth and an increase in the overall survival of mice with hepatocellular carcinoma Hepa1–6. Together with these effects, AFP-EVs reshaped the tumor microenvironment by attracting CD8^+^ T-lymphocytes to tumor sites and causing an increase in the levels of the immunostimulatory cytokines IFN-γ and IL-2 combined with reductions in immunosuppressive CD25^+^FoxP3^+^ T-regulatory cells and the levels of the immunosuppressive cytokines IL-10 and TGF-β (Lu et al., 2017).

Another tumor-associated antigen, HPV early antigen 7 peptide (E7_49–57_), which is the main target antigen in cervical cancer, was used to load murine EV-producing DCs (Chen et al., 2018). The treatment of TC-1 cervical cancer-bearing mice with E7_49–57_-loaded EVs induced an anti-tumor CTL response and activated potent protective and therapeutic immune responses *in vivo* (Chen et al., 2018).

Sources of multiple tumor antigens, such as tumor lysates or tumor total RNA, have advantages over single tumor antigens, since they contain a full set of tumor antigens and have the ability to activate a broad spectrum of polyclonal anti-tumor immune responses (Rizzo et al., 2014). Murine DC-derived EVs indirectly loaded with the lysate or chaperone-enriched lysate of GL261 mouse glioma cells have been obtained (Bu et al., 2015). DCs treated with EVs loaded with chaperone-enriched glioma lysate promoted the proliferation of CD4^+^ and CD8^+^ T cells and the activation of anti-tumor CTLs *ex vivo*, as well as the significant inhibition of tumor growth and prolonged survival of tumor-bearing mice. It was found that the chaperone-rich lysate contained at least four chaperone proteins including heat shock protein (Hsp) 70, Hsp 90, calreticulin, and glucose-regulated protein 94 (GRP94). Thus, chaperone-rich lysate is a superior source of tumor antigens for the loading of DCs and EVs in comparison with the lysate of tumor cells prepared using the conventional freezing–thawing method (Bu et al., 2015).

Human DC-derived EVs indirectly loaded with total tumor RNA or tumor lysate have been used to activate CTLs against human gastric adenocarcinoma BGC823 *ex vivo* (Guan et al., 2014). It was shown that DCs loaded with tumor RNA and EVs derived from these cells were more potent with respect to stimulating the proliferation of T cells and activated more efficient anti-tumor CTLs *ex vivo* in comparison with lysate-loaded DCs and their EV derivatives. However, all tested DCs or EVs inoculated in combination with T cells into tumor-bearing mice inhibited tumor growth with similar efficiency in a xenograft BGC823 tumor model (Guan et al., 2014).

The anti-tumor potential of T-cell vaccines stimulated with DC-derived EVs has been investigated (Wang et al., 2013). CD4^+^ T lymphocytes were primed with EVs isolated from DCs expressing HER2/Neu or HER2 tumor antigens *ex vivo* and used as anti-tumor immunotherapeutic vaccines. It was demonstrated that this immunotherapeutic approach provides significant results: efficient tumor-specific CTLs are activated and protective immunity is triggered in mice with HER2/Neu^+^ Tg1-1 breast cancer or HLA-A2^+^HER2^+^ B16-F10 melanoma.

Immunotherapeutic anti-tumor approaches have been widely investigated in tumor cells stably expressing ovalbumin (OVA), which are used as a useful model of tumor-specific antigens. OVA and the OVA-derived MHC I-restricted peptide, SIINFEKL, were used for the indirect loading of DC-derived EVs, and it was demonstrated that only OVA-loaded EVs, but not EVs loaded with the OVA-derived MHC I-restricted peptide, caused the induction of OVA-specific cytotoxic CD8^+^ T cells *in vivo*. The activation of CTLs was dependent on OVA-specific CD4^+^ T cells and B lymphocytes, since the full-length OVA protein contains both Th- and B-cell epitopes, whereas the SIINFEKL peptide is CD8^+^ T cell-specific. OVA-loaded EVs were superior in protecting the mice against B16-OVA tumor growth in comparison with peptide-loaded EVs (Naslund et al., 2013). A subsequent study by the same scientific group was devoted to comparing the ability of small- and large-sized EVs produced by OVA-loaded DCs to stimulate OVA-specific immune responses (Wahlund et al., 2017). Small-sized EVs (exosomes precipitated by ultracentrifugation at 100,000*g*) carried high levels of intact OVA inside and OVA peptides on their surface, whereas large-sized EVs (MVs pelleted by centrifugation at 10,000*g*) contained barely detectable levels of OVA. OVA-loaded small-sized EVs were shown to induce more potent OVA-specific T- and B-lymphocyte immune responses *in vivo* in comparison with large-sized EVs (Wahlund et al., 2017). It should be mentioned that small-sized EVs derived from immature DCs were shown to interact with T cells via CD80 signaling, inducing the secretion of Th1 cytokines (IFN-γ), whereas large-sized EVs induced Th2 cytokine synthesis (IL-4, IL-5, and IL-13) via CD40 signaling. Maturation of EV-producing DCs with LPS abolished the functional differences between small- and large-sized EVs: both types of EVs induced Th1-specific IFN-γ synthesis (Tkach et al., 2017).

DC-derived EVs have been indirectly loaded with a combination of model antigens, OVA and α-galactosylceramide (αGC), the latter of which is a ligand of invariant NKT cells (iNKT cells). Such EVs induced potent innate (NK and γδT lymphocytes) and OVA-specific T- and B-cell adaptive immune responses *in vitro* and *in vivo*. Using a mouse B16-OVA melanoma model it was demonstrated that DC-derived EVs loaded with OVA/αGC caused significant retardation of tumor growth, promoted infiltration of tumor tissues with antigen-specific CD8^+^ T cells, and increased the median survival time of tumor-bearing mice in comparison with those treated with a combination of soluble OVA and αGC (Gehrmann et al., 2013).

Undoubtedly, NK cells play a significant role in tumor immunosuppression; therefore, the potent anti-tumor potential of DC-derived EVs to activate NK cells and induce adaptive immunity is of great importance. EVs produced by non-loaded mouse DCs and EVs secreted by human DCs loaded with MAGE3 peptides have been used to treat mouse YAC-1 lymphoma and human advanced melanoma (stages IIIb and V) in phase I clinical trials. It was demonstrated that both mouse and human DC-derived EVs carried functional IL-15Rα receptors that could transpresent IL-15 to NK cells to stimulate their activation, proliferation, and IFN-γ synthesis *in vitro* and *in vivo*. Additionally, activation of NK cells was shown to occur via the interaction of NKG2D ligands (ULBP-1, MICA/B), carried on the surface of DC-derived EVs, with NKG2D receptors on NK cells (Viaud et al., 2009). NK cells can also be activated through the interaction of TNF receptors on NK cells with TNF ligands expressed on the surface of DCs and their EVs. It has been clearly demonstrated that even intact EVs produced by mature DCs carrying transmembrane TNF are able to activate NK cells and stimulate IFN-γ synthesis (Munich et al., 2012). Furthermore, these EVs were shown to express other TNF superfamily ligands, such as FasL and TRAIL, and cause the direct induction of apoptosis of B16 melanoma, KLN205 lung squamous cell carcinoma, and MC38 colon adenocarcinoma cells *in vitro* (Munich et al., 2012). It has also been demonstrated that DC-derived EVs can bind LPS and Pam3CSK4, ligands of toll-like receptors (TLR) 4 and TLR1/2, respectively. DCs loaded with such EVs were shown to upregulate the expression of transmembrane TNF, activate NK cells, and stimulate NK cells to secrete of IFN-γ (Sobo-Vujanovic et al., 2014).

The level of anti-tumor immune response triggered by DC-derived EVs directly depends on the degree of maturity of DCs and the type of maturation stimuli. The anti-tumor potential of EVs derived from murine DCs loaded with OVA or HOCl-oxidized B16-OVA cells that were matured with poly(I:C) (TLR3 ligand), LPS (TLR4 ligand), and CpG-B oligonucleotide (TLR9 ligand) have been compared in a model of B16-OVA melanoma *in vivo* (Damo et al., 2015). It was demonstrated that all types of EVs were able to significantly retard tumor growth. However, EVs derived from necrotic B16 cell-loaded DCs treated with poly(I:C) were the most efficient and induced robust activation of melanoma-specific CD8^+^ T cells in tumor-draining lymph nodes, spleen, and tumor tissues and recruited NK and NK-T cells to the tumor site, resulting in drastic inhibition of tumor growth and an increase in survival in tumor-bearing animals (Damo et al., 2015). Moreover, EVs produced by poly(I:C)-matured DCs loaded with HPV early antigen 7 markedly inhibited murine TC-1 cervical tumor growth and improved the survival rate of tumor-bearing mice (Chen et al., 2018). Thus, it has been revealed that the TLR3 ligand poly(I:C) is a favourable TLR agonist for DC maturation during antigen loading, which significantly increased the potential for anti-tumor immunity induced by antigen-loaded EVs, and could be suggested as a promising maturation stimulus for DC-derived EVs.

EVs produced by heat shock-exposed DCs and tumor cells have proven themselves as efficient immunotherapeutic vaccines. Tumor cells are characterized by the unusual overexpression of heat-shock proteins, such as HSP70, HSP90, and HSP72, which protect tumor cells from apoptosis induction (Ferrarini et al., 1992; Jäättelä et al., 1992). Thus, these heat-shock proteins can serve as tumor-specific antigens to activate anti-tumor immune responses. Heat-stressed EVs (HS-EVs) are commonly isolated from DCs or tumor cells that are exposed to hyperthermia at 42°C to 43°C for 1 to 4 h *in vitro* (Chen et al., 2011; Zhong et al., 2011; Wang et al., 2015) or from human tumor cells isolated following induction of hyperthermia in patients at 39°C for 1 h (Guo et al., 2018). HS-EVs contained high levels of HSP70 and HSP60, indicating significant immunotherapeutic properties (Zhong et al., 2011). Such HS-EVs promoted maturation of DCs, activated proliferation of T-lymphocytes, and induced tumor-specific immune responses *in vivo* (Zhong et al., 2011; Guo et al., 2018). HS-EVs were also shown to contain the chemokines CCL2, CCL3, CCL4, CCL5, and CCL20, which chemoattracted CD11c^+^ DCs and CD4^+/^CD8^+^ T cells into tumor tissues *in vivo* (Chen et al., 2011). DCs treated with HS-EVs were able to convert immunosuppressive T-regulatory cells to Th17 cells, contributing to the rejection of established prostate cancer in mice (Guo et al., 2018).

As it has been reviewed above, accumulated material on the potential of DC-derived EVs confirmed that these EVs are able to directly initiate strong anti-tumor innate and adaptive immune responses *in vivo*, protect experimental animals against tumors in prophylactic settings, and significantly reduce tumor growth and metastasis in therapeutic regimen.

Furthermore, the immunogenic potential of DC-derived EVs has been clearly demonstrated in phase I and II clinical trials (Escudier et al., 2005; Morse et al., 2005; Dai et al., 2008; Viaud et al., 2009; Besse et al., 2016) (see **Table 2C**). DC-derived EVs were shown to be safe for patients and to stimulate anti-tumor CTLs and NK cells. However, the actual clinical potential of DC-derived EVs to trigger anti-tumor immune responses, reduce tumor size and metastasis, and increase the survival time in patients remains undefined. It is hoped that DC-derived EVs will be more successful anti-tumor vaccines in comparison with DC-derived vaccines.

## Concluding Remarks—EVs Problems and Opened Questions

As mentioned above, great success is achieved on application of DC-targeted/-derived EVs to treat tumors in different murine tumor models and promising results are obtained in phase I and II clinical trials. Experimental data obtained to date points to similar or even superior ability of DC-targeted or DC-derived EVs to activate anti-tumor immune responses in comparison with classic DC-based vaccines. To enhance the anti-tumor and immunogenic potential of EVs, they are modified with a variety of molecules, such as tumor-associated antigens, tumor- or DC-targeted molecules, small non-coding regulatory RNA, and immunostimulatory molecules. Almost all methods of EV modification to possess anti-tumor properties are considered in the present review.

Nevertheless, some problems and opened questions on anti-tumor application of EV-based vaccines are still unsolved.

First, physiological activity of EVs is still poorly understood. In general, it is not yet entirely clear how EV-mediated paracrine regulation between cells occurs. The mechanism of selection of biologically active molecules (such as miRNA, cytokines, peptides, etc.) to EVs is not completely elucidated. Data on targeted delivery of natural EVs to specific cells and tissues are insufficient. Therefore, further comprehensive and in-depth investigations of biological properties of EVs are of immediate interest.

One of the main problems of EVs to investigate their biological properties and use them in clinical trials is inability of modern methods to isolate pure fractions of EVs without any mixture of residual subpopulations of other vesicles or even non-vesicular particles (Chulpanova et al., 2018). Hence, it is challenging to establish Clinical Good Manufacturing Practice (cGMP)-grade EVs preparations. Novel isolation methods are required to enrichment of the specific EVs subtypes. Therefore, better knowledge of specific markers of EVs subtypes is required.

Application of EV-based anti-tumor vaccines is associated with additional questions. How should dosing of inoculated EVs be determined? It is known that EV-mediated signaling is dose-dependent (Yu et al., 2007), so variation of EVs dose is able to impact on the balance between deleterious and therapeutic potential of administered EVs. In addition, does the route of administration (subcutaneous, intradermal, intravenous, etc.) impact the efficiency of EV-based antitumor therapy?

Additionally, to prepare sufficient amounts of therapeutic EVs, a big number of EV-producing cells and a huge volume of EV-containing condition media should be processed, that significantly complicates technological process and increases the price of potential antitumor EV-based vaccine. Highly scalable methods for mass production of EVs are required at all stages of the manufacturing process.

Finally, targeting of EVs to specific cells and tissues requires further optimization, as well as more efficient techniques for loading EVs with NAs, proteins, lipids should be developed.

In the near future these problems and questions will need to solve by enthusiastic multidisciplinary collaboration of molecular biologists, immunologists, biochemists, together with physicians to develop highly efficient next generation of EV-based anti-tumor vaccines.

## Author Contributions

OM analyzed published data and prepared the manuscript. AO prepared the part of the manuscript devoted to delivery of nucleic acids using extracellular vesicles. NM revised and corrected the manuscript.

## Funding

The present work was funded by the Russian Science Foundation (grant 17-74-10144) (OM), the Russian Foundation for Basic Research [grants 17-04-01136 (AO), 17-04-00999 (NM)], and the Russian State funded budget project of ICBFM SB RAS АААА-А17-117020210024-8 (NM).

## Conflict of Interest

The authors declare that the research was conducted in the absence of any commercial or financial relationships that could be construed as a potential conflict of interest.
